# Eleven years of student replication projects provide evidence on the correlates of replicability in psychology

**DOI:** 10.1098/rsos.231240

**Published:** 2023-11-08

**Authors:** Veronica Boyce, Maya Mathur, Michael C. Frank

**Affiliations:** ^1^ Department of Psychology, Department of Medicine, Stanford University, Stanford, CA, USA; ^2^ Quantitative Sciences Unit, Department of Medicine, Stanford University, Stanford, CA, USA

**Keywords:** metascience, replication, large-scale replication project, pedagogical replication, social psychology, cognitive psychology

## Abstract

Cumulative scientific progress requires empirical results that are robust enough to support theory construction and extension. Yet in psychology, some prominent findings have failed to replicate, and large-scale studies suggest replicability issues are widespread. The identification of predictors of replication success is limited by the difficulty of conducting large samples of independent replication experiments, however: most investigations reanalyse the same set of 170 replications. We introduce a new dataset of 176 replications from students in a graduate-level methods course. Replication results were judged to be successful in 49% of replications; of the 136 where effect sizes could be numerically compared, 46% had point estimates within the prediction interval of the original outcome (versus the expected 95%). Larger original effect sizes and within-participants designs were especially related to replication success. Our results indicate that, consistent with prior reports, the robustness of the psychology literature is low enough to limit cumulative progress by student investigators.

## 1. Introduction

Cumulative scientific progress requires empirical results that are robust enough to support both future empirical extensions and the construction of synthetic theories. Yet in psychology, some prominent individual findings have failed to replicate in multi-site replication attempts (e.g. terror management theory, [1,2]). One early large-scale replication project pegged the replication rate for findings in top-tier psychology journals at around 40% (RP:P; [3]). Low replicability has negative consequences for the field as a whole: when scientists attempt to build on published results, they stand a good chance of meeting with failure.

Addressing this issue requires a better understanding of the scope of the problem as well as the methodological and structural issues that might lead to replication failure (e.g. [4,5]). Estimating replicability in the literature is a key starting point, but, as we review below, there is limited consensus on what quantity exactly should be estimated.

Our viewpoint here is that one important estimand is the probability that a graduate student can identify a finding in the literature and replicate it successfully enough that they can build on that finding in subsequent work. Taking this perspective, our contribution is a new dataset of 176 replications of experimental studies from the social sciences, primarily psychology. These replications were conducted as individual course projects by students in a graduate-level experimental methods class between 2011 and 2022. We use this dataset to investigate the rate of replicability for such projects as well as the correlates of replication success.

### 1.1. What are we estimating when we measure replicability?

A few large-scale investigations have measured replication rates in samples of psychology studies. The first of these, RP:P, sampled roughly 100 studies from articles published in three top psychology journals in 2008 and distributed the studies across participating laboratories, finding an overall replication rate of around 40% [3]. The follow-up Many Labs studies investigated heterogeneity using short target studies that each compared two conditions. These study designs were not representative of the psychology literature as a whole, and due to the goal of measuring heterogeneity, they had large overall samples across multiple sites. Across Many Labs 1–3, only 29 of 51 target effects (57%) replicated [6,7]. Camerer *et al.* [8] included all 21 behavioural social science studies from *Nature* and *Science* from 2010 to 2015 that were feasible to replicate. They consulted original authors and had high power to detect even small effects; under these conditions and with this sample, the replication rate was around 60%.

While their sampling procedure and methods varied, these previous approaches to replicability have all focused on interpreting their results in terms of a potentially problematic estimand: the probability of a finding in the literature being truly replicable. Critics have pointed out that ‘true’ replicability may not be possible to estimate outside of a specific sample [9] or even time period [10].

Further, the methods used in these studies are not sufficient to yield an unbiased estimate of this quantity. In no case were studies randomly sampled from the literature; instead replication projects sampled from specific journals and adjusted the sample for feasibility concerns. These reasonable decisions further undermine the interpretation of the results from these studies as representing the proportion of true findings in the psychology literature as a whole.

Rather than aim for some measure of true replicability, perhaps we should contextualize the true estimand for replication efforts based on their methodologies and outcome measures. Through this lens, RP:P estimated the rate at which findings from relatively simple experiments published in a few well-known journals at a particular time could be replicated in a typical psychology laboratory. Many Labs estimated the rate at which well-known, two-condition findings replicate in very large samples. Camerer *et al.* [8] estimated which prestigious journal findings replicate when conducted in a highly resourced environment with expert involvement. All of these could be potentially desirable estimands.

But in practice, most scientific work is conducted by graduate students with limited time, limited budgets and limited access to experts. If students cannot replicate a finding under these circumstances, they cannot build on it in their own empirical or theoretical work. How replicable are findings in the literature for graduate students operating under these less-than-ideal conditions? We address this question here.

Our current sample of replications is selected based on what experiments students were interested in and wanted to replicate, with some filtering for feasibility. This sampling is not at all random. It reflects how scientists generally choose which experiments to build on: those that are interesting and relatively feasible given methodological and budgetary constraints.

In the current study, we estimate the probability of successful replication in this sample, with the goal of also identifying markers of when findings can (and cannot) support cumulative science. Our hope is to extend previous work that has attempted to find key correlates of replication success.

### 1.2. When do replications succeed?

Despite variation in the methods and outcomes used by large-scale replication studies, they are often aggregated together in analyses looking at the predictability of replication success. Prediction markets and elicitations have established that people can predict above chance what studies will replicate [8,11–13], but have not identified concrete predictors that differentiate replications from non-replications. Machine learning approaches trained on the available replications are also above chance at predicting replication success [14,15], though again the precise features relating to success are unclear.

In search of such features, RP:P examined correlates of replicability in the RP:P sample and found that studies in cognitive psychology (as opposed to social psychology) and studies with larger effect sizes and smaller *p*-values were more likely to replicate. Using these same data combined with a few other smaller samples, [16] examined statistical and demographic features of replication studies and identified larger sample sizes, larger effect sizes, and simple effects (as opposed to interaction terms) as predictive of replication.^1^

These approaches are fundamentally limited by the available data. Large-scale replications are arduous and expensive to run, so only a few large-scale replication datasets exist, and most analyses draw heavily on same small set of data points. In particular, the RP:P dataset is much discussed and reanalysed [19–22]—to the point that much of what we think we know about replicability may be overfit to the 100 studies included in RP:P.

Further, none of these studies focused on features of experimental design, such as within-participants designs or the use of repeated measures. There has been speculation that both of these factors should be linked to increased replicability due to their role in enabling increased statistical precision. Within-participants designs lead to more precise experimental estimates by allowing the estimation of correlated person-level variation across conditions; repeated measures allow for more precise estimation by averaging out measurement error. Both are often recommended by methodologists as part of good measurement practices, at least when they are feasible [23–25].

In sum, our current study examines the overall rate of replicability as well as the statistical and design features that predict replicability in a new sample of student replications.

## 2. Results

PSYCH 251 is a graduate-level experimental methods class in experimental psychology taught at Stanford University. During the 10-week class, each student replicates a published finding. They individually reimplement the study, write analysis code, pre-register their study, collect data (typically using an online crowd-sourcing platform), and write a structured replication report. Students in the course are free to choose studies related to their research interests, with the default recommendation being an article from a recent year of *Psychological Science*.

The sample of replicated studies reflects the variability of the literature, including studies from different subfields (and occasionally fields outside of psychology), with different experimental methods and statistical outcomes. We leveraged naturally occurring variability in this sample of replications to examine how different demographic, experimental design and statistical properties predict replication success.

These replications were all conducted on short time scales, within a constrained class budget. In some cases, the budget limited the number of participants who could be recruited, occasionally below what the original study included or what power analyses suggested. The replications had a median post-exclusion sample size that was 86% of the original sample size. In nearly all cases, replications were conducted online, with recruitment from Amazon Mechanical Turk (the default from 2011 to 2020) or Prolific (the default from 2021 to 2022). The goal was for replications to be as close as possible to the original, but budgetary constraints, inability to option original materials, and primarily using online samples meant that replications varied in their degree of closeness to the original. According to the schema from [26], 19% of studies were exact replications, 29% were very close replications, 44% were close replications and 8 were far replications (See Methods for more details on common deviations).

Many different measures can be used to define replication success of an individual statistical result [27–29]. However, whether a replication should be considered successful is not always dependent on only one statistical comparison between the two studies. Often in original papers, multiple statistical tests are cited in support of the claim that a pattern of results matches a particular theoretical expectation.

As our primary outcome, we chose to use a subjective replication score (coded by two independent raters—one typically at the time of project completion—with discrepancies resolved by discussion). Unlike statistical measures, subjective replication success accommodates studies with multiple important outcome measures that together define the pattern of interest. Further, this measure was applicable across the diverse range of statistical measures and reporting practices present in the sample.

**Figure 1.**
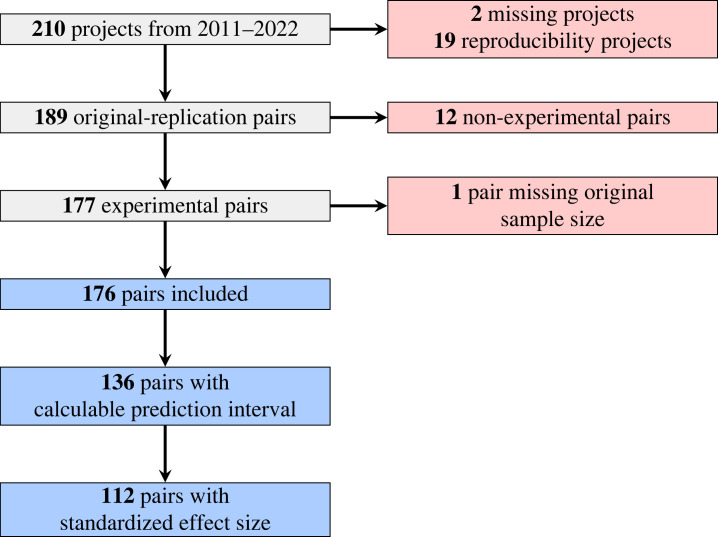
Of the 210 projects conducted for the class, 176 are included in our analysis, after excluding reproducibility projects (with no new data collection), non-experimental replications and missing projects. Of the 176, 136 report sufficient information to calculate prediction intervals, and 112 report enough to calculate standardized effect sizes.

As a complement to our primary subjective outcome, we also used two statistical measures of replication on the subset of the data where they were computable for the key statistic of interest (136 cases, see figure 1). We used *p-original*, the *p*-value on the null hypothesis that the original and replication statistics are from the same distribution, and *prediction interval*, a binary measure of whether the replication statistic fell within the prediction interval of the original statistic [29]. The prediction interval depends on the level of evidence of the original study; if the effect was marginal, the prediction interval could overlap zero; thus, a replication might fall within the predictive interval and be consistent with the original outcome, but not provide compelling evidence for the claimed effect. Conversely, large original effects with precise point estimates may have prediction intervals that do not overlap a smaller replication effect size, and thus would be inconsistent with the original outcome, even though researcher intuition might classify it as a success. Thus, these two statistical metrics each quantify the similarity between a key statistic in the original study and the replication, but they will not always match researcher intuitions on whether a study replicated.

### 2.1. Overall replication rate

Across the 176 studies, the average subjective replication score was 49%, which we can interpret as an overall subjective replication rate. In total, 45% (61/136) of replications had outcomes with point-estimates within the prediction interval of the original outcome. The median *p*-original value on the original and replication point-estimates coming from the same distribution was 0.03, representing the median probability that a replication study’s estimate would be at least as extreme as was actually observed, if in fact the replication and original were statistically consistent. The subjective replication scores were moderately correlated with both prediction interval (*r* = 0.36) and *p*-original (*r* = 0.27) values; prediction interval and *p*-original were highly correlated with each other (*r* = 0.73). As mentioned above, inconsistency between the subjective replication and numeric outcomes can occur in a few circumstances such as when there is a large precise original effect and a smaller, but clear replication effect; when there is a small, imprecise original effect and a smaller, null, or opposite replication effect; or when the key measure does not represent the overall pattern of the results.

**Figure 2.**
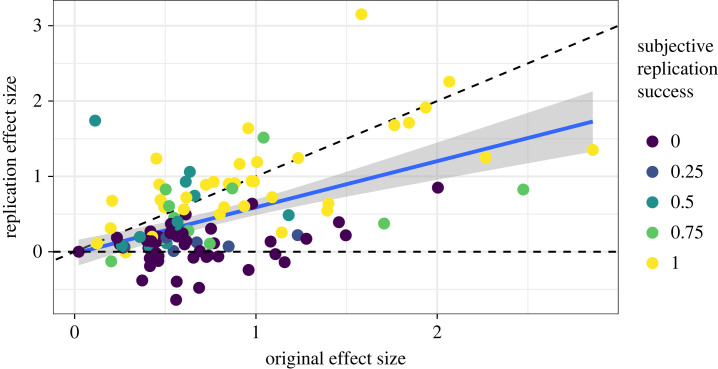
Relationship between effect size of the original study, effect size of the replication study and subjective replication success rating, for those studies where effect size was applicable (*N* = 112).

Figure 2 shows the relationship between original standardized effect size, replication effect size and subjective replication score. Some studies replicated with similar size effects to the original, and others failed to replicate, with replication effect sizes near zero.

On average, there was a diminution of effect sizes from original to replication. This pattern of results is consistent with the results of RP:P [3]. Specifically, the median original effect size (in standardized mean difference (SMD) units) was 0.61 (interquartile range: 0.46–0.98), and the median replication effect size was 0.28 (0.09–0.76), for a median difference of 0.31 (0.04–0.66). Among the pairs with a subjective replication score of 1 (*N* = 38 with standardized effect sizes), the median original effect size was 0.92 (interquartile range: 0.52–1.4), and the median replication effect size was 0.89 (0.57–1.24), for a median difference of 0.03 (−0.16−0.33). Thus, for the highest subjectively rated replications, effect sizes for original and replication were similar in magnitude on average, with some replications having larger effects than their originals.

Some multi-site replication projects have found heterogeneity in effect sizes across replication sites [18,30,31]. As a test of sensitivity to heterogeneity, we assumed that the level of heterogeneity in hypothetical multi-site replications of our sampled articles was the same as the average-level heterogeneity found in [31] in prior multi-site replications in psychology (*τ* = 0.21 in SMD units). Under this assumption, 63% (71/112) of replication effect sizes are distributionally consistent with the original effect size. However, more work on understanding heterogeneity is needed to understand what levels of heterogeneity to expect across different implementations of the same experiment, and how considerations of heterogeneity should impact interpretations of both novel results and replication results.

### 2.2. Bivariate correlates of replication success

We investigated what features of the original study and replication were correlated with replication success, with the goal of being able to identify potential markers of replicability. We chose a set of predictor variables based on the correlational results of RP:P [3], our own intuitions of experimental design factors that might impact replication success, and some covariates related to how close the replication was; table 1 has descriptive statistics of the distribution of values for these features in the dataset, and a full description of these features is given in the Methods.

**Table 1.** Descriptive properties of the replication dataset. For categorical features, counts and percentages are given. For continuous features, the median value and interquartile range are given. *N* = 176 except for original effect size and *p*-value where *N* = 112. See Methods for full description of how these values were coded.

feature	summary
subfield: cognitive psychology	62 (35%)
subfield: social psychology	70 (40%)
subfield: other psychology	24 (14%)
subfield: non-psychology	20 (11%)
open data	52 (30%)
open materials	83 (47%)
switch from in-person to online	94 (53%)
original authors at Stanford	16 (9.1%)
within participants design	80 (45%)
single vignette (one item/condition)	78 (44%)
number of trials	6 (1, 60)
publication year	2015 (2011, 2017)
original sample size	101 (40, 181)
replication sample size	59 (31, 125)
ratio of replication/original sample sizes	0.86 (0.41, 1.05)
original effect size (SMD)	0.61 (0.46, 0.98)
original *p*-value	0.0001 (0.0000, 0.0069)

Many predictors correlated with subjective replication success using unadjusted Pearson correlations (table 2). Predictors of higher replicability included within-participants designs, larger numbers of trials per participant, and the original study having openly accessible data. Predictors of lower replicability included single-vignette studies (those with only one experimental stimulus per condition), classification as social psychology, and study pairs where the original study was in-person and the replication switched to online.

**Table 2.** The unadjusted Pearson correlations between each individual predictor and the subjective replication score. See Methods for how these variables were coded.

*r*	*p*	predictors
0.333	0.000	within participants design (versus between participants)
0.182	0.015	log number of trials
0.150	0.047	open data
0.080	0.294	non-psychology (versus cognitive psych)
0.075	0.322	other psychology (versus cognitive psych)
0.064	0.399	publication year
0.002	0.979	open materials
−0.027	0.725	Stanford affiliation of original authors at time of replication
−0.047	0.536	log ratio between replication and original sample sizes
−0.108	0.155	log original sample size
−0.158	0.037	switch to online for replication (versus same modality for original and replication)
−0.246	0.001	social psychology (versus cognitive psych)
−0.267	0.000	single vignette (versus multiple items/inductions per condition)

**Figure 3.**
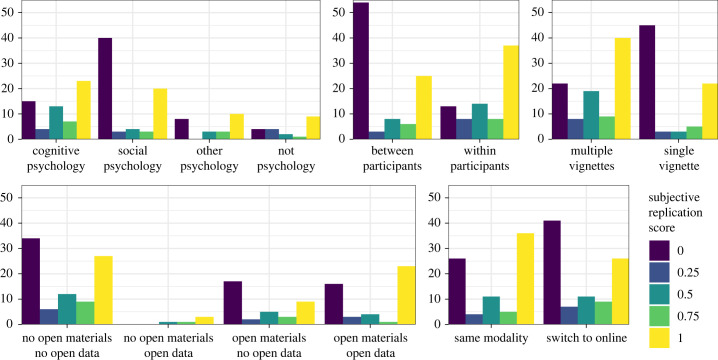
Distribution of subjective replication scores within categories. Bar heights are counts of studies.

**Figure 4.**
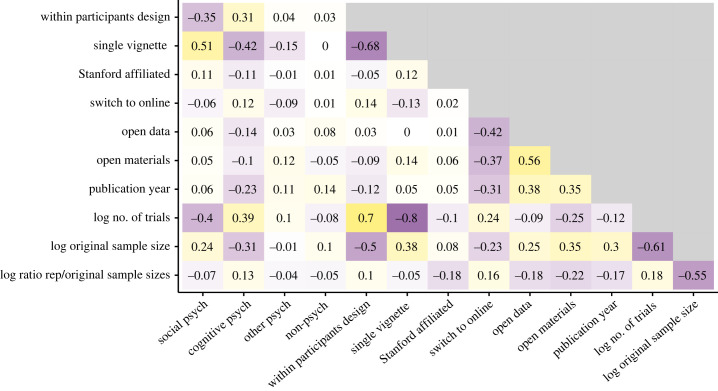
Correlations between predictor variables. See Methods for descriptions of how each variable was coded.

Distributions of study outcomes across some of these properties are shown in figure 3. Both social and cognitive psychology studies were well represented in the replication sample, and the cognitive psychology studies replicated more often than social psychology studies (mean subjective replication scores: 0.58 versus 0.36). Within and between participants designs were both common, and within-participants designs replicated more often than between-participants designs (mean scores: 0.65 versus 0.36). Studies with multiple vignettes replicated more often than single vignetted studies (mean scores: 0.59 versus 0.36). However, there were strong correlations among these experimental features as well as between these experimental features and specific subfields (figure 4).

Studies with open data, which almost always also had open materials as well, tended to replicate more than studies without open data. Nearly all replication studies were conducted online, but original studies were split between using in-person and online recruitment. Replications that switched to online were less likely to replicate than those that had the same modality as the original (generally both online, in a few cases both in-person). While online studies in general show comparable results to studies conducted in person [32], switching the modality does decrease the closeness of the replication, and some studies done in person may not have been well adapted (e.g. inductions might have been weaker or instructions might have been insufficient). These factors—open materials, open data, and online samples—may have all contributed to the closeness of the replications. However, they are also practices that have increased over time, and so these effects may partially reflect temporal trends. Nonetheless, publication year only very weakly correlated with replicability, but does correlate strongly with online samples and openness (figure 4).

### 2.3. Joint evaluation of the predictors of replicability

While a number of features show individual correlations with the subjective replication score, many correlate with one another. To determine which predictors were the strongest, we modelled subjective replication score as an ordinal outcome using ordinal Bayesian regression models with a logit link function, regularized using a horseshoe shrinkage prior [33]. This model estimates odds ratios representing the association of the predictor with having a higher versus lower subjective replication score. We first ran models using all of the original-replication pairs (*N* = 176), but without original effect size and original *p*-value as predictors, as they were uncodable for some pairs. We next ran models including all predictors, but on only the subset of data where all predictors were available (*N* = 112).

**Figure 5.**
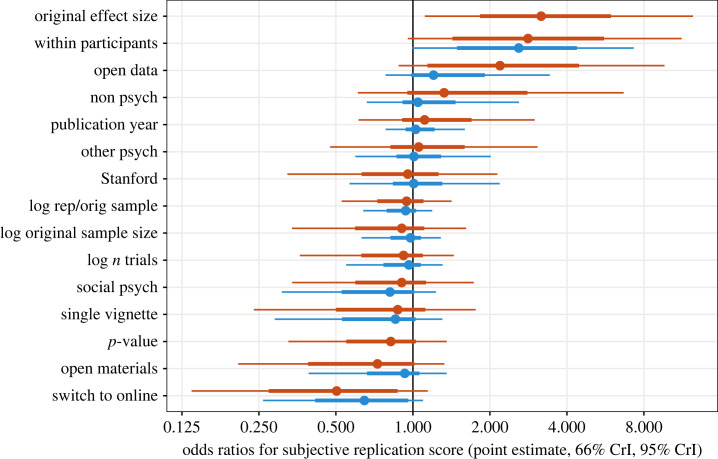
Odds ratios and corresponding 95% credible interval (CrI) on the likelihood of having a higher subjective replication score as a function of the independent variable. Estimates from a model of all original-replication pairs (*N* = 176) are shown in blue, and from a model of all pairs with full statistical information (*N* = 112) are shown in red. A value of 1 indicates no association, greater than 1 indicates an association with higher replication scores and less than 1 an association with lower replication scores.

Coefficient estimates from the two ordinal models predicting the subjective replication scores are shown in figure 5. Due to a large number of predictors coupled with a small and noisy dataset, there is much uncertainty around the coefficients even with strong regularization. The general directions of coefficients are consistent with the effects of the predictors in isolation.

Within-participants designs stand out as the strongest correlate of replicability in the model with all the data (odds ratio (OR) = 2.59, 95% credible interval (CrI) =[1,7.3]). In the model with all predictors, but less data, within-participants designs remain strong (OR = 2.89, 95% CrI = [0.96, 11.24]), and standardized effect size is also substantially related to subjective replication score (OR = 3.33, 95% CrI=[1.11,12.44]). Both effects are robust to a sensitivity analysis including only studies with close replications and matching statistical tests (within-participants OR = 3.58, 95% CrI=[0.91,19.57]; effect size OR=7.22, 95% CrI=[1.24,47]).

We also ran models predicting our secondary outcome measures: a logistic model predicting whether the replication effect was within the prediction interval of the original effect, and a linear model predicting what the *p*-original was between the replication and original. Both these models had more uncertain estimates. While the credible intervals were wide, the general patterns of predictor direction and relative strength were similar to the subjective replication models (see electronic supplementary material for results from all models). The strongest predictors for prediction interval were still within-participants designs (OR = 1.78, 95% CrI=[0.86,8.3]) and studies with larger effect sizes (OR = 0.98, 95% CrI=[0.56,1.63]).

## 3. Discussion

Non-replications pose a problem for scientists who want to build on the empirical results in the literature, but the limited numbers of replications and limited research into specific predictors of replication failure mean that the reasons for non-replications are not well understood.

Here, we take a functional approach to assessing replicability, framing both our methods and interpretation around the idea of whether work can be repeated by an early-career scientist. We took advantage of 11 years of graduate student replication projects to study both the overall level of replication success and the correlational predictors of replication in a previously unused dataset. In line with previous results, we found a 49% replication rate, with some studies showing effect sizes similar to the original and others much smaller. Within-participants designs, work in the subfield of cognitive psychology, and the original and replication both using online samples stood out as the strongest correlates of replication success. As many of these predictors interrelate with one another, we ran regularized regressions with all the predictors at once. Due to our small sample, model estimates were uncertain, but within-participants designs and large original effect sizes were the strongest predictors.

We do not interpret our non-replications as indicating the original results were false positives: presumably some were and some were not. There are many possible reasons for the non-replications in this sample. In some cases, the problem may be with the replication, such as too few participants, many exclusions for failed attention checks, or participants speeding through the study. When these issues are diagnosed, they can suggest possible ways to ‘rescue’ the replication by increasing the sample or changing the interface, without altering the underlying experiment; thus, while the replication did not succeed, after some troubleshooting, students may still be able to extend the work in the future. In other cases, there were *a priori* reasons to distrust the original study, such as exclusion criteria that seemed *post hoc* or high-order interaction terms with a small sample. That said, not all scientists recognize the same factors as potential indications of low power or questionable research practices; students conducting these replications generally expected them to succeed. In many—perhaps most—non-replications in our sample, it was unclear why the results failed to replicate.

Our results are limited by the sample of studies we included, which are limited in number and may not be representative of the studies of interest to psychologists as a whole. Further, our predictor variables were not manipulated, so they cannot be interpreted as causing (non-)replication, but only as correlational markers. Some of the correlates are most easily interpreted as being about the original study, and others reflect the closeness of the replication to the original.

For instance, while within-participants designs are more likely to replicate than between-participants designs, this predictor could also be related to the types of experiments that tend to be run in each design. Additionally, as [24] notes, within-participants designs can lead to practice effects, carry-over of treatments, and critically, sensitization effects, where participants reason about the contrast between conditions and respond differently due to that reasoning. Sensitization effects are a threat to internal validity, and could be replicable even in the absence of the effect itself being replicable. Given the strong relationship of within-participants designs to replicability, slightly more scepticism and critical reading of between-participants designs may be warranted, but this correlation, by itself, does not mean scientists should prefer within-participants designs.

Large-scale replication studies are costly and arduous. The batch of replications presented here were pedagogical replications, done as part of a class. Trainees must learn experimental methods, and conducting replications as part of methods classes serves several purposes: it enables students to learn to do experiments in a supportive context, it often leads to more useful results than if students designed their own experiments from scratch, and it creates a resource for studying the literature [34–37]. We believe that this kind of pedagogy has an important role to play in improving methodological practices in psychology more broadly. The tools and workflows of rigorous, replicable science cannot simply be mandated: they have to be learned.

## 4. Methods

Our pre-registration, code and coded data are available at https://osf.io/xwn9m/ [38].

At the onset of this project, all authors had some familiarity with the dataset. M.C.F. taught PSYCH 254/251; M.M. and V.B. had each been students in the class. The coding of the data primarily took place before the pre-registration was finalized; the only way for us to know what variables were actually codable and what forms of missingness were present in the data was to code it. Thus, the pre-registration was a pre-registration of the analyses and a ‘locking-in’ of the coding scheme. V.B. coded both the predictor variables and was one of the coders of outcome variables, but these were coded in separate passes through the data.

### 4.1. Deviations from pre-registration

The descriptive statistics and bivariate correlations between predictor and outcome variables were not pre-registered.

In the pre-registration, our research questions mentioned publication journal, but due to the distribution of journals, journal was not a predictor in the pre-registered model plan (or the actual models). Our pre-registration stated that we could not include students' names or reports in the released dataset; we ended up letting students opt-in to associating their names with their projects and having their replication report included. Our pre-registration stated that any errors found would be noted; errors are noted in the data spreadsheet of the materials.

### 4.2. Dataset

The dataset of replication projects comes from class projects conducted in PSYCH 251 (earlier called PSYCH 254) a graduate-level experimental methods class taught at Stanford by M.C.F. from 2011 to 2022. This class is commonly taken by first-year graduate students in psychology and related disciplines, and it has been a requirement of the Psychology PhD since around 2015. Each student chose a study to replicate, implemented the study, wrote analysis code, pre-registered their replication, ran the study and turned in a structured final report including methods, analytic plan, changes from the original study, confirmatory and exploratory analyses, and discussion of outcomes. Students were encouraged to do experimental replications, but some students chose to replicate correlational outcomes or do computational reproducibility projects instead. We cannot include the full student reports for confidentiality reasons, but we include over 50 reports that we received permission to share and the template given to students at https://osf.io/xwn9m/ [38].

Students were free to choose what study to replicate; the recommended path for students who did not have their own ideas was to pick an interesting study from a recent year of *Psychological Science* (this led to 80 *Psych Science* articles in the replication sample, 45% of all studies).

Replications varied in how close they were to the original; while the goal was to replicate the original as closely as possible, some deviations were sometimes necessary. Student reports contained a section listing changes from the original. In response to a reviewer, we attempted to code studies using the classification scheme from [26].

Common reasons for replications being very close instead of exact were a switch from in-person to online and lack of access to the original instructions. (It was not always possible to tell if students had access to original wording and presentation style of materials.) Common reasons for replications being only close included recreating materials when the original materials were not available and changing materials to fit the audience (e.g. switching from UK to US English). (It was not always possible to determine if students had the original materials; in some cases students mentioned trying to obtain materials but did not indicate if they had succeeded.) Less common reasons for replications being only close included changing the number of trials per participant or reducing training periods.

Far replications were rare, but were primarily due to a couple of deviations. In some cases an original study had a specific population (e.g. high schoolers or hiring managers), and the replication was on a convenience population. The other main reason was changing the language of materials to English when cultural or linguistic factors were potentially relevant to the construct of interest.

Other common changes were changing or reducing demographic surveys (which were used only in exploratory or secondary analyses, if at all) and removing secondary measures that were not part of the analyses being replicated. We did not consider these as reducing the degree of closeness.

Four of the replication projects were included in RP:P, and 10 were previously reported in [37] (which reported 11 student replications from the 2015–2016 class; one of those was excluded from the current sample for being non-experimental).

### 4.3. Coding procedure

We relied primarily on student reports to code the measured variables for the replications. We supplemented this with spreadsheets of information about projects from the time of the class and the original papers.

#### 4.3.1. Measures of replication success

Our primary replication outcome was experimenter- and instructor-rated replication success. The subjective replication success was recorded by the teaching staff for the majority of class replications at the time they were conducted. Where the values were missing they were filled in by M.C.F. on the basis of the reports. For all studies, replication success was independently coded by V.B. on the basis of the reports. Where V.B.’s coding disagreed with the staff/M.C.F.’s code, the difference was resolved by discussion between V.B. and M.C.F. (26% of studies). Subjective replication scores were coded on a [0, 0.25, 0.5, 0.75, 1] scale.

This subjective replication outcome was chosen because it already existed, could be applied to all projects (regardless of type and detail of statistical reporting) and did not rely solely on one statistical measure. As a complement, we also identified a ‘key’ statistical test for each paper (see below for details), and if possible, computed *p*-original and prediction interval at this statistic, following [29]. *p*-original was a continuous measure of the *p*-value on the hypothesis that the original and replication samples come from the same distribution. Prediction interval was a binary measure of whether the replication outcome fell within the prediction interval of the original outcome measure.

#### 4.3.2. Demographic properties

We coded the subfield of the original study as a four-way factor: cognitive psychology, social psychology, other psychology and non-psychology. For each paper, we coded its year of publication, whether it had open materials, whether it had open data, and whether it had been conducted using an online, crowd-sourced platform (i.e. MTurk or Prolific).

#### 4.3.3. Experimental design properties

We coded experimental design on the basis of student reports, which often quoted from the original methods, and if that did not suffice, the original paper itself. To assess the role of repeated measures, we coded the number of trials seen per participant, including filler trials and trials in all conditions, but excluding training or practice trials.

We coded whether the manipulation in the study was instantiated in a single instance (single vignette). Studies with one induction or prime used per condition across participants were coded as having a single vignette. Studies with multiple instances of the manipulation (even if each participant only saw one) were coded as not being single vignette. While most studies with a single vignette only had one trial and vice versa, there were studies with a single induction and multiple test trials, and other studies with multiple scenarios instantiating the manipulation, but only one shown per participant.

We coded the number of participants, post-exclusions. We coded whether a study had a between-participants, within-participants, or mixed design; for the analysis, mixed studies were counted as within-participants designs. In the analysis, we used a log-scale for number of participants and numbers of trials.

#### 4.3.4. Properties of replication

We coded whether the replication was conducted on a crowd-sourced platform; this was the norm for the class projects, but a few were done in-person. For analysis, we coded this into a variable indicating if the recruitment platform changed between original and replication. This grouped the few in-person replications in with the studies that were originally online and stayed online in a ‘no change’ condition, in contrast with the studies that were originally in-person with online replications.

We coded the replication sample size (after exclusions). This was transformed to the predictor variable log ratio of replication to original sample size.

As a control variable, we included whether the original authors were faculty at Stanford at the time of the replication. This was to account for potential non-independence of these replications (e.g. if replicating their advisor’s work, students may have access to extra information about methods).

We made note of studies to exclude for sensitivity analyses, due to not quite aligned statistics, extremely small or unbalanced sample sizes, or a student choosing a key statistical measure that was not of central importance to the original study.

#### 4.3.5. Determination and coding of key statistical measure

For each study pair, we used one key measure of interest for which we calculated the predictor variables of *p*-value and effect size and the statistical outcome measures *p*-original and prediction interval. If the student specified a single key measure of interest and this was a measure that was reported in both the original paper and replication, we used that measure. If a student specified multiple, equally important, key measures, we used the first one. When students were not explicit about a key measure, we used other parts of their report (including introduction and power analysis) to determine what effect and therefore what result they considered key. In a few cases, we went back to the original paper to find what effect was considered crucial by the original authors. When the measures reported by the student did not cleanly match their explicit or implicitly stated key measure, we picked the most important (or first) of the measures that were reported in both the original and replication. These decisions could be somewhat subjective, but importantly, they were made without reference to replication outcomes.

Whenever possible, we used per-condition means and standard deviations, or the test statistic of the key measure and its corresponding degrees of freedom (e.g. *t*-test, *F*-test). We took the original statistic from the replication report if it quoted the relevant analysis or from the original paper if not. We took the replication statistics from the replication report.

We then calculated *p*-values, effect sizes, *p*-original and prediction intervals. We chose to recalculate *p*-values and effect sizes from the means or test statistic rather than use reported measures when possible because we thought this would be more reliable and transparent. The means and test statistics are more likely to have been outputted programmatically and copied directly into the text. By contrast, *p*-values are often reported as less than 0.001 rather than as a point value, and effect size derivations may be error prone. By recording the raw statistics we used, and using our available code to calculate other measures, we are transparent, as the test statistics can be searched for in the papers, and all processing is documented in code.

In some cases, *p*-values or effect sizes were not calculable either due to insufficient reporting (e.g. reporting a *p*-value but no other statistics from a test) or key measures where *p*-values and effect sizes did not apply (e.g. principal component analysis (PCA) as measure of interest). Where studies reported beta estimates and standard errors or proportions, standardized effects sizes are not an applicable measure, but we were still able to calculate *p*-original and prediction interval.

We separately coded whether the original and replication effects were in the same direction, based on raw means and graphs. This is more reliable than the statistics because *F*-tests do not include the direction of effect, and some students may have flipped the direction in coding for betas or *t*-tests. In the processed data, the direction of the effect of the replication was always coded consistently with the original study’s coding, so a positive effect was in the same direction as the original and a negative effect in the opposite direction.

In regression analyses, we used standardized mean difference and log *p*-value as predictors.

### 4.4. Modelling

All original-replication pairs (except for one) had codable demographic and experimental features, while fewer pairs had codable effect sizes on some consistent scale, and fewer still had codable *p*-values and SMD effect sizes. Thus, we had more predictor variables and outcome variables for some original-replication pairs than for others, but which variables were codable was monotonic. To take full advantage of the data, we ran a series of models, with some models having fewer predictors, but more data, and others having more predictors, but less data.

We ran a model predicting the subjective replication score on the basis of demographic and experimental predictors on the entire dataset. We ran two models predicting *p*-original and predicting whether the replication was in the prediction interval from demographic and experimental predictors on the subset of data where we had *p*-original and prediction intervals. Then, on the smaller subset of the data where we had effect sizes and *p*-values, we reran these three models with those as additional predictor variables.

The subjective replication scores were coded on [0, 0.25, 0.5, 0.75, 1], and we remapped these to 1–5 to run an ordinal regression predicting replication score. We ran logistic regressions predicting prediction interval and linear regressions predicting *p*-original.

We used a horseshoe shrinkage prior on the fixed effect coefficients because we had a lot of predictors compared with the amount of data [33]. All models included random slopes for predictors nested within year the class occurred to control for variation between cohorts of students. We did not include any interaction terms in the models. All numeric predictor variables were *z*-scored after other transforms (e.g. logs) to ensure comparable regularization effects from the horseshoe prior. The priors we used were horseshoe (3) for betas, normal (0, 0.5) for standard deviation of random slopes and lkj (1) for correlations between random slopes. Models were run in brms [39].

As a secondary sensitivity analysis, we examined the subset of the data where the statistical tests had the same specification, the result was of primary importance in the original paper (i.e. not a manipulation check), and there were no big issues with the replication.

Results from these models not reported in the main paper are reported in the electronic supplementary material.

## Data Availability

Our pre-registration, code and coded data are available at https://osf.io/xwn9m/. Supplementary material is available online [40].

## Footnotes

1 While most approaches to correlates of replicability have been correlational, experimental approaches can be used to test potential interventions [17,18]. Experiments can be very valuable as tests of specific causes of non-replication, but they are expensive and time-consuming to conduct, and not all factors affecting replication success can be manipulated experimentally.
